# The use of qualitative methods to inform Delphi surveys in core outcome set development

**DOI:** 10.1186/s13063-016-1356-7

**Published:** 2016-05-04

**Authors:** T. Keeley, P. Williamson, P. Callery, L. L. Jones, J. Mathers, J. Jones, B. Young, M. Calvert

**Affiliations:** Centre for Patient Reported Outcomes Research, Institute of Applied Health Research, University of Birmingham, Birmingham, England; Department of Biostatistics, MRC North West Hub for Trials Methodology Research, University of Liverpool, Liverpool, England; Midwifery and Social Work, School of Nursing, University of Manchester, Manchester, England; Department of Psychological Sciences and MRC North West Hub for Trials Methodology Research, University of Liverpool, Liverpool, England

**Keywords:** Core outcome sets, Qualitative research, Delphi, Methodology, Clinical trial

## Abstract

**Background:**

Core outcome sets (COS) help to minimise bias in trials and facilitate evidence synthesis. Delphi surveys are increasingly being used as part of a wider process to reach consensus about what outcomes should be included in a COS. Qualitative research can be used to inform the development of Delphi surveys. This is an advance in the field of COS development and one which is potentially valuable; however, little guidance exists for COS developers on how best to use qualitative methods and what the challenges are. This paper aims to provide early guidance on the potential role and contribution of qualitative research in this area. We hope the ideas we present will be challenged, critiqued and built upon by others exploring the role of qualitative research in COS development.

This paper draws upon the experiences of using qualitative methods in the pre-Delphi stage of the development of three different COS. Using these studies as examples, we identify some of the ways that qualitative research might contribute to COS development, the challenges in using such methods and areas where future research is required.

**Results:**

Qualitative research can help to identify what outcomes are important to stakeholders; facilitate understanding of why some outcomes may be more important than others, determine the scope of outcomes; identify appropriate language for use in the Delphi survey and inform comparisons between stakeholder data and other sources, such as systematic reviews. Developers need to consider a number of methodological points when using qualitative research: specifically, which stakeholders to involve, how to sample participants, which data collection methods are most appropriate, how to consider outcomes with stakeholders and how to analyse these data. A number of areas for future research are identified.

**Conclusions:**

Qualitative research has the potential to increase the research community’s confidence in COS, although this will be dependent upon using rigorous and appropriate methodology. We have begun to identify some issues for COS developers to consider in using qualitative methods to inform the development of Delphi surveys in this article.

## Background

Randomised controlled trials (RCTs) typically provide robust evidence, which can be used to inform clinical practice and health policy [1]. The outcomes measured within a RCT allow the benefits (or harms) associated with an intervention to be quantified. Outcomes measured in RCTs need to be useful and relevant to a range of stakeholders including patients, clinicians, policy-makers and regulatory agencies [2, 3]. Outcomes are often identified, chosen and specified a priori by the trial management team (traditionally, researchers and clinicians), sometimes with input from patient and public contributors [4].

The use of numerous varying trial outcomes across a research field or clinical area can be problematic. First, this can reduce the ability of systematic reviewers to synthesise results. The most accessed Cochrane reviews of 2009 all reported problems with heterogeneity of outcomes [5], while similar problems were found in an analysis of the ClinicalTrials.gov database [6]. Second, lack of an accepted standard can lead to reporting bias, based on the significance of the findings [7–9]. Furthermore, outcomes that are selected solely by researchers or clinicians may not hold relevance for other stakeholders, such as patients, carers or other decision-makers.

These problems can be addressed through the development of a core outcome set (COS) for use in a clinical area or research field. A COS is a standardised collection of outcome domains that should be reported in all controlled trials within a research area [10]. Trialists are not restricted solely to these outcomes and can use additional outcomes to those in the core set; therefore, a COS marks the basic requirement for which outcomes need to be measured and reported in all studies in a field [11]. Furthermore, COS development is typically focussed initially on *what* to measure with subsequent consideration needed of *how* to measure those core outcomes. In this paper we use the term ‘outcome’ to refer to outcome domains.

The rate of development of COS has increased over the last 10 years, to the point where close to 20 new COS were published in 2013 [12]. Core outcome sets have been developed for use in a wide variety of clinical specialties [13], including cancer, rheumatology, neurology and cardiorespiratory research; for use with different populations, such as adults and children; and for use specifically in pharmaceutical or surgical research. The development of COS is attractive to funders such as the National Institute for Health Research (NIHR) and others, as it increases the chance that the ‘value of their investments will be greater than the sum of the reports’, through the increased ability to synthesise and compare results, as well as a greater assurance the that outcomes used in funded studies will be of relevance to stakeholders [14].

The methods used in COS development exercises are important as they may influence the final COS [3]. Development of a COS can comprise several phases, often starting with a systematic review of the published literature to identify what outcomes have been measured in previous trials or studies in a clinical area. This may generate a ‘long list’ of candidate outcomes for a COS. Consensus methods, such as simple face-to-face meetings, nominal group techniques and, increasingly, the Delphi survey, may then be used to reach agreement about which outcomes are ‘core’ [3, 13]. The Delphi is often followed by a consensus meeting of key stakeholders to agree the final COS. Qualitative research can be used in several of these phases, but our main focus in this paper is to outline the use of qualitative research to inform Delphi surveys in COS development.

A Delphi survey is a sequential process through which the opinions of participants are sought, usually anonymously [11]. Participants in a Delphi survey do not interact directly; rather, after the completion of each round of questionnaires, the collated group responses are fed back to participants. In this way, equal weight is given to all those who participate and the risk of an individual or group of individuals being overly influential or dominant in the process is reduced [15].

Of the 227 COS studies published up to the end of 2014, 38 (17 %) included the use of Delphi surveys, while the rate of use in ongoing studies appears to be higher still. The majority of COS studies using Delphi survey will use a modified rather than a traditional Delphi. In a ‘traditional’ Delphi the outcomes of potential importance would be identified solely in the first round of the Delphi through the use of an open text question [16]. In modified Delphi surveys in COS development, a ‘long list’ of outcomes is identified prior to the Delphi survey, often, as noted above, through a systematic review of outcomes measured in previous trials.

However, a list of outcomes identified through such systematic reviews may largely reflect outcomes that researchers have thought important to measure, particularly where trials predate the recent emphasis on patient and public involvement (PPI) in the design. Patients, carers and healthcare professionals might differ from researchers in what outcomes they see as important. Relying solely on systematic reviews of previous trials may lead to outcomes that are important to patients and other stakeholders being overlooked. Trialists need to have confidence that the perspectives of all relevant stakeholder groups have been heard and that their views of important outcomes are incorporated into the Delphi and, depending on the results of the Delphi, into the final COS. To address this COS developers have recently incorporated qualitative research into the development process to help ensure that the outcomes in a COS are important to the whole community of stakeholders, including patients [13]. Often this has involved qualitative data collection methods such as focus groups and one-to-one interviews with patients, carers and healthcare professionals [17, 18]. However, little methodological guidance or precedent is available about how qualitative research can best be used to inform this component of COS development [19, 20].

### Aim

This paper has two aims. First, we discuss the potential roles for which primary qualitative research may be used in the pre-Delphi stage of the development of a COS. Second, we highlight considerations for conducting primary qualitative research in the pre-Delphi stage of a COS development based on our experiences of using qualitative research in three COS development processes (Table 1).

**Table 1:** Description of studies used to inform this paper

	PARTNERS2 [30]	CONSENSUS [31]	mOMEnt [32]
Study title	Core outcome sets for use in effectiveness trials involving people with bipolar disorder and schizophrenia in a community-based setting	CONSENSUS – squamous cell CarcinOma of the oropharyNx: late phaSE cliNical trialS; core oUtcomeS	mOMEnt – management of Otitis Media with Effusion in cleft palate: protocol for a systematic review of the literature and identification of a core outcome set using a Delphi survey
Qualitative data collection method	Focus groups and one-to-one semi-structured interviews, with prompts to cover key discussion points. Topic guide used as an aide memoire and iteratively updated	One-to-one or three-way semi-structured interviews with patients and their carers. The topic guide comprised prompts to ensure key topics were explored and was iteratively developed	Conversational style interviews with parents including prompts to discuss topics identified from relevant literature. Developmentally appropriate interviews with children
Participants	Bipolar disorder and schizophrenia service user and their carers and healthcare and research professionals working in this area	UK and US patients treated for oropharyngeal squamous cell carcinoma (a type of head and neck cancer) and their carers	Parents of children with non-syndromic cleft palate (including cleft lip and palate) between 0 and 11 years of age, who had a current or past diagnosis of OME, and children themselves aged 6–11 years
Ethical approval	Ethical approval for the study has been sought and granted from the National Research Ethics Service (NRES) West Midlands – Edgbaston (reference number 14/WM/0052)	Ethical approval for this study was sought and granted in the UK by the Liverpool Central Research Ethics Committee (reference 12/NW/0708). Approval at the University of Texas MD Anderson Cancer Center (Houston, TX, USA) was provided by the Institutional Review Board (protocol number 2013–0285)	Ethical approval for the qualitative interviews with parents and children was sought and granted by the National Research Ethics Service – NRES North East Committee – Greater Manchester East (reference 11/NW/0586)

The PARTNERS2 COS development is part of a larger NIHR-funded project titled: PARTNERS2: Development and pilot trial of primary care-based collaborative care for people with serious mental illness [33]. The mOMEnt COS development was part of a larger NIHR-funded project titled: The management of Otitis Media with Effusion in children with cleft palate (mOMEnt): a feasibility study and economic evaluation [21]. The CONSENSUS study was conducted by Aoife Waters as part of a PhD, supported by the Medical Research Council via the North West Hub for Trials Methodology Research

This paper is not intended to be prescriptive; rather it looks to provide early guidance, which we hope will be challenged, critiqued and built upon by others exploring the role of qualitative research in COS development. We conclude by identifying a number of areas where future research may be beneficial to COS developers.

## Results and Discussion

The discussions and advice provided in this paper are based upon the experience of authors in developing three COS in different research areas, with differing participants and using different qualitative data collection methods. Box 1 summarises the COS developments which have been drawn upon.

### The role of qualitative research in core outcome set development

As noted above, qualitative research can be used in the pre-Delphi stage of a COS development for a number of purposes:

1. Identification of outcomes that are important to stakeholders
  Qualitative research allows COS developers to explore the views of patients, healthcare professionals and other stakeholders in order to inform the development of a ‘long list’ of potential outcomes. The discursive nature of qualitative research enables participants to explain important features of conditions and treatments in their own terms rather than requiring them to engage with a discourse of ‘outcomes’, which is likely to be unfamiliar to those outside clinical research. If conducted appropriately and rigorously the qualitative research should afford stakeholders the opportunity to explore and identify outcomes of importance. In turn, this should promote COS developers’ confidence that *all* potentially relevant outcomes have been included in the first round of the Delphi survey. In doing so, stakeholders have the opportunity to set the agenda and potentially identify outcomes that researchers may not have anticipated. For example in mOMEnt, participants emphasised psychological as well as social consequences of impaired hearing, including frustration and behavioural problems in children. This contrasted with the limited reporting of these outcome domains in the literature; of the 49 papers identified in the mOMEnt systematic review, two included outcomes related to psychosocial development and six included outcomes related to behaviour [21]
2. Facilitate understanding of not only which outcomes are important, but crucially why they are important
  Qualitative research with patients, carers and other stakeholders can allow a greater understanding of why an outcome is of importance. For example, in PARTNERS2 employment was found to be an important social outcome for many people recovering from serious mental illness, and was identified in both the literature review and primary qualitative research. However, the qualitative research allowed us to understand that suitable employment was more important than employment per se and that its importance stemmed from the financial security, meaningful role, structure to the day or the connectedness which attending a workplace can allow. By illuminating the context in this way, qualitative research can ensure that meaningful and accurate outcomes are taken forward into the Delphi survey and brief yet informative descriptions to accompany outcome names are developed to help to ensure that participants can interpret a Delphi survey. Furthermore, findings from qualitative research may help to inform discussion at the later consensus meeting (particularly if there are disagreements among stakeholders), facilitating agreement on the final set
3. Determining the scope of outcomes
  Qualitative research may also allow the scope of outcomes to be defined in a way which holds most relevance to stakeholders in the Delphi. Take the example of quality of life, which is a frequently measured and reported outcome in trials. There are numerous different conceptualisations of quality of life that can be measured, varying from broad definitions, such as global quality of life, capability or well-being, to narrower definitions, such as health-related quality of life or disease-specific quality of life. Furthermore, within health-related quality of life, there are subdomains of physical functioning and psychological well-being. Qualitative research can be used to delimit the scope of the domain and ensure that the breadth of domain taken forward to the Delphi is appropriate
4. Identification of appropriate language for use in a Delphi survey
  The language used to describe outcomes in clinical trial publications may differ markedly from the language used by patients, carers and other stakeholders. Qualitative research that identifies and describes outcomes using participants’ own narratives can help COS developers to label and describe outcomes in ways that make sense to the stakeholders participating in the Delphi survey. This is important to ensure a Delphi survey is accessible. For example, based on qualitative findings the research team may choose to describe the outcome of isolation as ‘feeling cut off and distant from friends’ or the outcome of aggression as ‘getting wound up, angry or lashing out’
5. Comparison with other stakeholder data or alternative sources of outcome data
  Finally, outcomes derived from qualitative data collected from different stakeholder groups, such as service users, carers and healthcare professionals can be compared within the study to understand areas of discordance. When used in combination with a systematic review of current outcomes this can allow the COS developers to assess whether the ‘standard’ outcomes used in trials in that research area are inclusive of the outcomes that stakeholders think should be measured. Or, whether the outcomes currently used in a research area may be missing important domains and should be supplemented when taken into round 1 of the Delphi survey. For example, in PARTNERS2 ‘symptoms’ was identified as an important outcome by service users and carers, healthcare professionals and through the review of literature. However, a clear area of discordance was found whereby service users emphasised ‘living with existing symptoms’ as important, while the healthcare professional data and the review data focused on ‘symptoms’ reduction’. In this case, both outcomes are being taken into the Delphi, with correct terminology and descriptions used to ensure the differences in the two domains were evident to Delphi participants.

### Deciding when qualitative research might not be needed?

As discussed above, qualitative research may allow the views of a broad range of stakeholders to be included in the development process of a COS and facilitate a move away from researcher-only selected outcomes. However, qualitative research can be resource-intensive; both in terms of time and costs and the requirement for specialist input from qualitative experts. COS developers may want to consider whether such work is needed in the particular clinical area for which they are developing the core set. Developers may want to consider the following points: What is the level of PPI in the research area? If there has been a high level of PPI input into relevant trials and research studies, it may be reasonable to assume that outcomes in the area already reflect the perspectives of these stakeholders, although this may be challenged on the grounds that PPI is not *research*. Developers might also want to explore whether there are existing qualitative datasets that could help to identify outcomes of importance to stakeholders. If relevant studies have been conducted in the area, it may be possible for these data to inform the COS development through secondary analysis. How challenging is the phrasing of outcomes in the Delphi thought to be? For populations or areas where participants are likely to be particularly sensitive to the wording of outcomes, such as children or end of life care, the extra investment may be beneficial to ensure the wording is acceptable and appropriate. These are some points which developers may want to consider; however, this is not an exhaustive list and other considerations may be important.

### Challenges in using qualitative research to inform the development of core outcome sets

#### Which stakeholders to include?

It is important to consider which stakeholders to include as participants in qualitative research to best inform COS development. Being a participant in a qualitative study demands no prior knowledge of concepts such as ‘outcomes’, and no understanding of research processes or the rationale for COS (see section below on discussing outcomes). Therefore, qualitative data collection methods are appropriate when working with stakeholder groups such as patients, carers and healthcare professionals for whom such topics may be unfamiliar.

Patients have valuable first-hand experience of living with the illness and receiving treatments and knowledge about which outcomes are important to them. Healthcare and health research professionals may have experience of treating a number of patients or observing a number of research projects and, therefore, understand how an illness manifests itself in different individuals or the different treatment effects in individuals. Other stakeholders such as carers, who are typically spouses or family members, can provide useful perspectives as ‘involved witnesses’.

While our experience indicates that patients, carers and professionals tend to identify some similar outcome domains as important, there have also been some differences. For example, in PARTNERS2 when talking about physical health outcomes patients identified broad areas such as weight gain and reduced physical activity; whereas professionals talked about specific clinical outcomes, such as diabetes and blood pressure. Or, when discussing social outcomes, such as being able to participate in a work environment, healthcare professionals identified the ability to work as an important outcome; whereas patients and carers identified subtly different outcomes of participation in work that is appropriate to their condition (e.g. flexible working), and participation in a role that made them feel valued, as important outcomes.

There are indications from the broader literature that the differences we found between the outcomes that stakeholders identify (and the added value of including patients and carers) are widespread in this type of study. Qualitative studies have found that patients may prioritise different outcomes to healthcare professionals [22, 23] and may also identify additional important outcomes [24].

### Sampling

The pre-Delphi stage of the development of a COS needs to identify outcomes that are relevant to all stakeholders. A number of studies of qualitative outcomes have reported difficulty accessing a broad range of participants [17, 22, 24]. Therefore, it is important that the sampling strategy facilitates access to patients, carers, professionals and other participant groups who have experience of the illness for which the COS is being designed. If a key aim of pre-Delphi qualitative research is to ensure no outcomes are overlooked, there is a strong case for using a sampling strategy designed to identify a maximum variation sample, as this would be more likely to identify the wide range of outcomes of interest.

Purposive sampling can be used to recruit heterogeneous maximum variation samples, where people differ by select characteristics [25]. This allows participants to be selected based upon characteristics which might be anticipated to influence the outcomes they perceive as important [26, 27]. Parents of children of all ages in the mOMEnt study identified hearing as an important outcome. However, their concerns about hearing differed between parents of preschool children (0–4 year-olds) who focused on speech and language; parents of young primary school children (5–7 year-olds) who emphasised effects on social interaction; and parents of older primary school children (8–11 year-olds) who were concerned about social interaction and educational performance [21]. These differences highlight the importance of including variation in a sample, in this case diversity of age and development of children.

Qualitative samples are normally smaller in size than quantitative samples, as quantification of incidence is not the focus of this research. Rather the purpose is to collect rich data that allows in-depth exploration and understanding of different research questions [28]. Normally there will come a point of diminishing return when new interview or focus group data cease to contribute to the analysis, and the research team will decide to stop data collection (the point of conceptual saturation). In the PARTNERS2 interviews with healthcare and research professionals this was noted after 14 interviews, with a further two interviews conducted to check that data saturation had been reached. A larger sample size may be required if particular diversity is needed in some characteristics. For example, as well as sampling participants from both the US and UK, the CONSENSUS study aimed to include a diverse group of patients in terms of sociodemographic, disease and treatment characteristics and, therefore, recruited over 30 patients and their carers. The mOMEnt study included a range of three cleft malformation types (palate only or in combination with either unilateral or bilateral cleft lip) and four treatment pathways (ventilation tubes, hearing aids, both, or watchful waiting) resulting in a qualitative sample of 37 children.

### Discussing trial outcomes

Qualitative research in the early phases of COS development will be focused on identifying outcome domains that are important to participants. Our experience suggests that the concept of an outcome can be rather obscure and challenging for patients, carers and other stakeholders to engage with. Patients and carers cannot be expected to be in a position to engage meaningfully with questions such as ‘what outcomes do you think we should measure in a trial of treatment for your illness?’ In the mOMEnt study parents did not respond readily to the notion of ‘outcome’. Therefore, parents were prompted to consider what they thought an intervention should achieve (Table 2). Despite this two parents did not distinguish between process (for example ‘good aftercare’) and outcomes. In the PARTNERS2 we also found that some healthcare professionals, researchers and commissioners also struggled to discuss outcomes directly. Our experience is likely to be reflective of challenges experienced by the broader research community, with a number of studies reporting similar challenges [17, 22].

**Table 2:** Questions and prompts used by authors to discuss outcomes

Discussions with patients	Discussions with healthcare/researcher professionals
PARTNERS2 ‘I would like you to think about how your mental health problems have changed your life and what you have lost because of them.’ ‘This time rather than thinking about what you have lost, I would like you think about what your goals are in living with your symptoms.’ ‘Since your diagnosis and treatment has life changed for you? In what ways has life changed?’ CONSENSUS ‘What’s a good day like for you? What’s a day like which is not so good’ ‘What would you say your priorities are in life at the moment? What would you have said if I’d asked that question before your illness and treatment?’ mOMEnt *–* Discussion with parents: ‘What do you think grommets (VTs) or hearing aids (HAs) should do for a child with glue ear?’ mOMEnt *–* Discussions with children: ‘What was “good” and “not so good” about VTs or HAs?’	PARTNERS2 ‘How does schizophrenia/bipolar disorder affect a person’s life? What do they lose?’ ‘What outcomes are you/should we looking to achieve when delivering care or support to people with bipolar disorder/schizophrenia?’ ‘What are you looking to improve in the person’s life?’ ‘Are different outcomes important to patients at different stages in their illness? At different stages in their health? Controlled versus stable? Diagnosis versus later management?’

To address this, careful consideration needs to be given to how outcomes are going to be elicited and discussed when designing qualitative research [29]. Normally this planning would involve consultation with relevant patient groups in order to inform the design of the research. Further consideration and consultation will be needed when developing the topic guide or interview schedule, when planning the prompts to be used and when iteratively developing these over the course of semi-structured interviewing to expand and explore participants’ accounts. Encouraging a discussion of how an illness has affected a person’s life, which parts of their life they may perceive to have lost and what things they hope to gain through treatment/care was found to be a fruitful way of approaching the discussion in all three of the studies used as examples in this paper. In CONSENSUS one-to-one interviews allowed patients to provide a chronological narrative of their lives as they underwent treatment and beyond. Over the course of their interviews patients spoke of how outcomes that were important early in treatment sometimes differed to those that became important at later stages. Interviews for the mOMEnt study commenced by inviting parents to tell the story of their child’s otitis media with effusion (OME) (or ‘glue ear’). These accounts provided narratives of the context of experiences of the condition and interventions and included implicit references to outcomes. As the interview progressed the participants were asked to discuss outcomes more explicitly. While in PARTNERS2 participants were encouraged to think back over how their illness had changed their lives and to discuss their goals in living with their condition. Later in the interview participants were encouraged to think about these changes and goals in terms of research outcomes. These may be reflective of similar approaches taken by other studies. For example, a qualitative study by Allard et al. to identify key outcomes for children with neuro-disability reported discussing outcomes by asking parents and carers about ‘aspects of health’ and using a visual aid in the discussion with children [17]. Similarly a qualitative study building the basis for a COS in rheumatoid arthritis asked patients about how they know when a intervention is working, what ‘returning to normal’ meant to them and what makes them feel well [22]. For all studies used as examples herein, allocating time to these early discussions in focus groups and interviews helped to identify outcomes of relevance and provided the basis for later discussions about which of the points discussed they felt were relevant to measure as outcomes in a research setting.

### Focus groups or interviews?

If the purpose of qualitative research prior to a Delphi survey is to identify a complete list of outcomes which may be important to stakeholders, then a data collection method that allows the patient’s journey to be understood may be most effective. However, if the purpose is to define the scope of the outcomes or the language, then an approach that allows convergences and divergences between different stakeholders to be identified may be most appropriate. However, often the objective of pre-Delphi qualitative research is to inform both a complete list and increase understanding of outcomes, which may call for a mix of qualitative data collection methods. Focus groups and one-to-one interviews are two ways in which qualitative data can be collected. These two methods of data collection have important differences which need to be considered when identifying outcomes in COS development.

In a one-to-one interview, data are generated through an interaction between the interviewer and the participant. A semi-structured format helps to ensure that the most important aspects are covered, while allowing the participant flexibility to explore concepts important to them. As described above this may involve participants giving an account of their illness and treatment experience, which researchers can interpret to identify outcomes which are important to patients.

In a focus group, data are generated through an interaction between the participants which is facilitated by the researcher. Participants are in a position to listen, discuss, agree, question or clarify points that are raised by other participants in the group. This synergistic discussion aims to facilitate participants in exploring outcomes which are important to them or the people they care for. Group discussion can help patients to see how their experiences differ to those of other participants in the groups and thereby help to identify outcomes which are important to them, or to challenge outcomes which are not important to them. However, there are drawbacks too. The logistics of completing groups can be challenging. Just as some people will dislike the idea of participating in an individual interview and prefer being part of a group, others may perceive a group discussion as intimidating and inhibitive. Additionally, a typical focus group involving 8–9 participants and lasting 90–120 minutes provides each individual with an average of only 10–15 minutes of speaking time, which can constrain the range of outcomes discussed.

Our experience of using focus groups in COS development indicates that while outcomes were discussed in depth, fewer outcomes were identified and understanding the patient journey and outcomes of importance at different stages was difficult. To address this challenge in PARTNERS2 we used a number of methods to collect non-verbal data, where participants were given the opportunity to write down outcomes of importance to them on slips of paper or ‘post-it’ notes. These data were then either used to inform discussion later in the focus group or were collected solely as written data. In some instances this exercise was designed to hide the identity of the note’s author to allow sensitive outcomes to be identified and subsequently discussed without embarrassment or inhibition.

### Analysis of data

When analysing the data from a qualitative study to support COS development, a focus must be maintained upon the particular purpose of the research. If, as described above, the main purpose of the research is threefold (to identify outcomes, define the scope of outcomes and identify common language) this must be reflected in the analysis. In many cases analytical approaches that code, label and index data will facilitate the process of identifying relevant outcome domains for the Delphi. Paying attention to, and maintaining the language of, the study participants will allow identification of common language. This should be part of an interpretive process whereby analysts consider the data as a whole in identifying relevant and understandable outcomes.

In the CONSENSUS study, for example, the coding, labelling and indexing of data, allowed the identification of the fact that patients tended to talk at length about the impact of treatment on aspects of their quality of life and how in contrast, survival was often mentioned only in passing or indirectly. One interpretation might be that survival was less important to these patients than aspects of their quality of life. However, considering the data and the interview as a whole the CONSENSUS team’s interpretation was that issues of life and death were difficult for patients to talk about. In the context of interviews where patients were describing the months of unpleasant treatment that they had endured to improve their chances of surviving the illness, the importance of survival did not need to be laboured.

### Future research

The use of qualitative research in the development of COS is increasing. This paper has described the potential benefits of qualitative research, indicated some of the challenges faced and provided examples of methods which may help to overcome them. The advice and guidance provided in this paper, which is not intended to be prescriptive, is based largely on the authors’ experiences of using qualitative research in the context of wider COS development projects. A better understanding of the role and contribution of qualitative research in COS development will depend on future methodological research. The following areas are identified as in particular need of such research.

#### Methods of data collection

More knowledge is needed on the differences in data collected from one-to-one interviews versus those collected from focus groups. As noted above our experience suggests that differences may arise; however, the nature and impact of differences on what is learnt and the associated resource use is not clear without further exploration. By reflecting on the use of qualitative data collection methods in COS development exercises to date, future research can be designed to assess whether interview and focus group data yield the same depth of meaning and understanding about stakeholder preferred outcomes and the extent and implications of any differences. Of course this cannot be considered in isolation from the points below.

#### Discussing outcomes

The way that outcomes are introduced to research participants and the framing of the discussion that follows will likely have a notable impact upon data collected. Research into the best ways to discuss outcomes with patients, carers and healthcare professionals – and whether to avoid overt discussions of ‘outcomes’ altogether – would help to ensure that participants are able to fully contribute to COS development process [29].

#### Analysing data

It is essential to understand the approaches to qualitative analysis that will be most informative for COS development. The need to go beyond a simple cataloguing of outcomes to form a deeper understanding of what participants wanted from treatments was identified in each of the COS development examples provided here.

#### Sampling

Understanding the impact of different sampling techniques is essential. As noted above, based on our experience, maximum variation sampling seems to be most likely to identify potentially important outcomes. However, confirmation of this and the potential effects of convenience or opportunistic sampling is vital.

#### Use of existing qualitative research

There is a large and expanding qualitative research literature on a wide range of different conditions and treatments and there are likely to be several such studies in particular disease areas that could potentially contribute to COS development, or even avoid the need to collect new qualitative data, which can be resource-intensive. Where qualitative datasets are available, secondary analysis of these may similarly negate the need for primary data collection, although research is needed to examine the extent to which such data, which will likely have been collected for very different purposes, can be used to inform COS development. Future research about how to usefully incorporate these data into COS development is of importance.

## Conclusion

The use of qualitative research in the pre-Delphi stage of COS development is a novel methodological advance which brings a number of potential benefits. These benefits all relate to the primary goal of ensuring that all stakeholder perspectives are represented in the final COS, whether through identification of outcomes, understanding the importance of outcomes or identifying patient and carer language. Our experience suggests that with these benefits come a number of challenges. This paper suggests a number of potential methodological solutions, which we hope will be investigated further by researchers in this field.

## Abbreviations

COS: core outcome set
PPI: patient and public involvement
RCT: randomised controlled trial
